# Regulatory Requirements for Interchangeable Biosimilar Designation

**DOI:** 10.1007/s43441-025-00897-6

**Published:** 2025-11-16

**Authors:** Praveen J. Samy, Morgane C. Mouslim, Charles L. Bennett, Antonio J. Trujillo

**Affiliations:** 1 Department of International Health, Johns Hopkins Bloomberg School of Public Health, Baltimore, MD USA; 2 The Hilltop Institute, University of Maryland, Baltimore County, 1000, Hilltop Circle, Baltimore, MD USA; 3 Department of Health Management and Policy, University of Michigan, Ann Arbor, MI USA; 4 College of Pharmacy, University of South Carolina, Columbia, SC USA

## Abstract

**Background:**

In 2019, the US Food and Drug Administration (FDA) finalized guidance for designating interchangeable biosimilars requiring pre-approval phase III clinical trials to evaluate safety when reference and biosimilar formulations are interchanged. In June 2024, Draft FDA Guidance on interchangeability relaxes FDA approval criteria focusing on analytic rather than clinical findings. This study examines US trends in interchangeable biosimilar approvals between 2019 and 2025, manufacturer strategies, and regulatory timelines.

**Methods:**

We used the FDA Purple Book archive (September 2025) to identify interchangeable biosimilars, associated Biologic License Application (BLA) numbers, manufacturers, reference products, and FDA approval dates. FDA submission dates were obtained from FDA approval letters. We analyzed trends by year, manufacturer, product, and product class to assess patterns in approval timing and strategy.

**Results:**

Since 2020, the FDA has approved 26 interchangeable biosimilars, with 19 approved between January 2024 and September 2025. Six are adalimumab biosimilars. Manufacturers employed two strategies: concurrent submission for interchangeability (17 cases) or delayed pursuit (9 cases). Average approval timelines decreased from 798 days for applications dated in 2020 to 364 days for applications dated in 2024. All interchangeable biosimilars completed pre-approval switching studies.

**Conclusions:**

Concurrent interchangeable designation is now the predominant regulatory pathway. The FDA June 2024 Draft Guidance, removing switching studies requirement for interchangeability might cut biosimilar development costs and development timelines. These changes, alongside provider and patient education, could enhance biosimilar uptake and reduce biologic drug costs.

## Introduction

In 2019, the Food and Drug Administration (FDA) issued guidance to enable approval of interchangeable biosimilars, allowing pharmacy-level substitution without prescriber authorization [1]. By 2024, 46 states permitted biosimilar substitution without physician consent. June 2024 Draft FDA Guidance proposes to remove requirements to conduct formal clinical switching trials, focusing instead on analytics. This study analyzes approval trends, timelines, and manufacturer strategies to inform policy and market development.

## Methods

Data were obtained from the FDA Purple Book archive (September 2025) [2] including Biologic License Application (BLA) numbers, biosimilar names, manufacturers, reference products, and approval dates. Submission dates for BLAs were sourced from FDA approval letters. Trends were analyzed by year, manufacturer, and reference product to assess evolution of markets and regulatory impact.

## Results

**Fig. 1:**
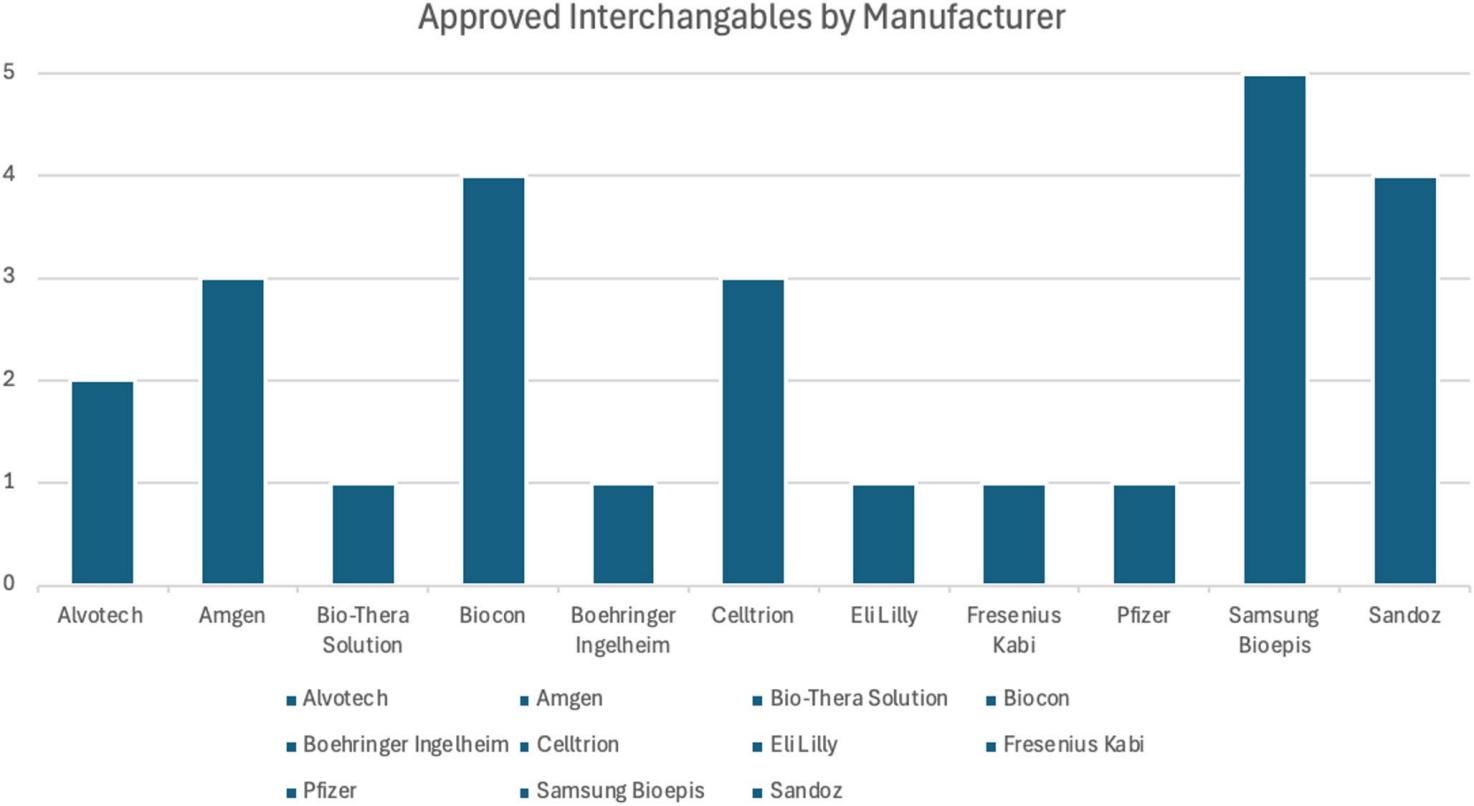
Number of Interchangeable biosimilar approvals, by manufacturer 2016–2025

Since 2016, approvals for interchangeability have largely favored major manufacturers. Samsung Bioepis, Sandoz, and Biocon lead with five, four, and four approvals, respectively followed by Amgen and Celltrion with three each (Fig. 1). Two dominant company strategies emerged: some sought interchangeability concurrent with their BLA approval (17 applications), while others delayed pursuing interchangeability (9 applications), possibly to assess market potential. FDA interchangeable approvals have increased over time. Annual approvals rose from 2 in 2021 to 15 in 2024 (Table 1), while the average review time decreased by 433 days, from 798 days for drugs that applied in 2020 to 364 days for drugs that applied in 2024 (Table 2). A significant proportion of interchangeable biosimilars received multiple formulation approvals. Of the 26 approved since 2020, 19 were cleared for two or more dosage forms or delivery methods, yielding 79 distinct product approvals. There were 11 total reference products and the reference drug with the most approved interchangeable biosimilars as of September 2025 is ustekinumab with seven followed by adalimumab with six. (Table 3).

**Table 1:** The number of interchangeable drugs approved by the FDA year. 2021–2025

Year	Approved interchangables
2021	2
2022	2
2023	3
2024	15
2025	4

**Table 2:** The average number of days between BLA submission and FDA approval per year of application. 2020–2024

Application year	Average time for approval (days)	Number of interchangeables
2020	798.00	4
2021	683.25	4
2022	455.60	5
2023	386.20	10
2024	364.33	3

**Table 3:** Number of FDA approved interchangeable biosimilar drugs by reference drug

Refrence drug	Approved Interchangables
Adalimumab	6
Aflibercept	2
Denosumab	2
Eculizumab	1
Etanercept	1
Insulin aspart	1
Insulin glargine	1
Omalizumab	1
Ranibizumab	2
Tocilizumab	1
Ustekinumab	7

## Discussion

Given the high cost of biologics, interchangeable biosimilars represent an opportunity to reduce healthcare costs and improve access to essential therapies. Despite the trends shown above, the U.S. biosimilar market remains underdeveloped compared to Europe. Since 2006, the EMA has approved 92 interchangeable biosimilars, compared to the FDA’s 26 since 2020 [3, 4]. Despite accelerated FDA timelines, approvals still often exceed one year, reflecting requirements for inspections, data review, and switching studies. Streamlining pathways for lower-complexity biosimilars (e.g., filgrastim, insulin, growth hormone) through a tiered system could encourage more manufacturers to enter the market. Granting biosimilar and interchangeable status at the same time and removing switching study requirements could further reduce delays while maintaining safety. Yet, uptake may be limited if incentive payments from PBMs favor reference products.

Aligning U.S. regulations with European standards would speed up approvals, lower costs, and expand treatment options. These steps could help realize the full potential of interchangeable biosimilars to improve care and reduce costs. While updated guidance could decrease costs and development timelines, it will also be imperative that that providers and patients are adequately educated on the safety and availability of interchangeable biosimilars. Patients and providers may not be aware of the availability of interchangeables, or may be wary of them [5]. Without proper education, providers and patients may request pharmacies to only dispense the reference drug rather than the interchangeable product. Next, without clarifying the distinction between “strength versus potency” in new 351 K filings for biosimilars and interchangeables, barriers to use of interchangeable biosimilars will occur because of safety concerns that arise when same strength products have different doses. New pharmacovigilance methods should be implemented to minimize clinician and patient safety concerns. Lastly, regulatory predictability is also essential for biosimilar uptake to occur seamlessly.

## Data Availability

All data were obtained from the FDA purple book.
